# Generational Differences in the Orientation of Time in Cantonese Speakers as a Function of Changes in the Direction of Chinese Writing

**DOI:** 10.3389/fpsyg.2012.00255

**Published:** 2012-07-26

**Authors:** Hilário de Sousa

**Affiliations:** ^1^Max Planck Institute for PsycholinguisticsNijmegen, Netherlands; ^2^SINOTYPE, Centre de recherches linguistiques sur l’Asie orientale, École des hautes études en sciences socialesParis, France

**Keywords:** time, space, script direction, Chinese script, Cantonese

## Abstract

It has long been argued that spatial aspects of language influence people’s conception of time. However, what spatial aspect of language is the most influential in this regard? To test this, two experiments were conducted in Hong Kong and Macau with literate Cantonese speakers. The results suggest that the crucial factor in literate Cantonese people’s spatial conceptualization of time is their experience with writing and reading Chinese script. In Hong Kong and Macau, Chinese script is written either in the traditional vertical orientation, which is still used, or the newer horizontal orientation, which is more common these days. Before the 1950s, the dominant horizontal direction was right-to-left. However, by the 1970s, the dominant horizontal direction had become left-to-right. In both experiments, the older participants predominately demonstrated time in a right-to-left direction, whereas younger participants predominately demonstrated time in a left-to-right direction, consistent with the horizontal direction that was prevalent when they first became literate.

## Introduction

Time is often described using spatial expressions cross-linguistically (e.g., Traugott, 1978; Lakoff and Johnson, 1980; Gibbs, 1994; Moore, 2006). This is perhaps because both time and space are dimensional, and they are both very prominent in discourse, as evidenced by how often space and time are encoded grammatically in languages, with, for instance, spatial deictics, tense, and spatio-temporal uses of switch-reference (e.g., de Sousa, 2006). Since space is directly perceivable by the external senses, it provides convenient apparatus for describing time, which is not directly perceivable by external senses. It has also been claimed that space does more than simply provide apparatus for describing time: people’s conception of space influences their conception of time (e.g., Boroditsky, 2000; Boroditsky and Ramscar, 2002; Casasanto and Boroditsky, 2008). One oft-cited spatial-linguistic factor that influences people’s conception of time is script direction (e.g., Tversky et al., 1991; Casasanto and Lozano, 2006; Núñez and Sweetser, 2006; Casasanto and Bottini, 2010; Fuhrman and Boroditsky, 2010; Bergen and Chan Lau, 2012). With the aim of testing this latter theory, the author conducted two experiments in Hong Kong and Macau with literate Cantonese speakers of various ages^1^.

**Figure 1:**
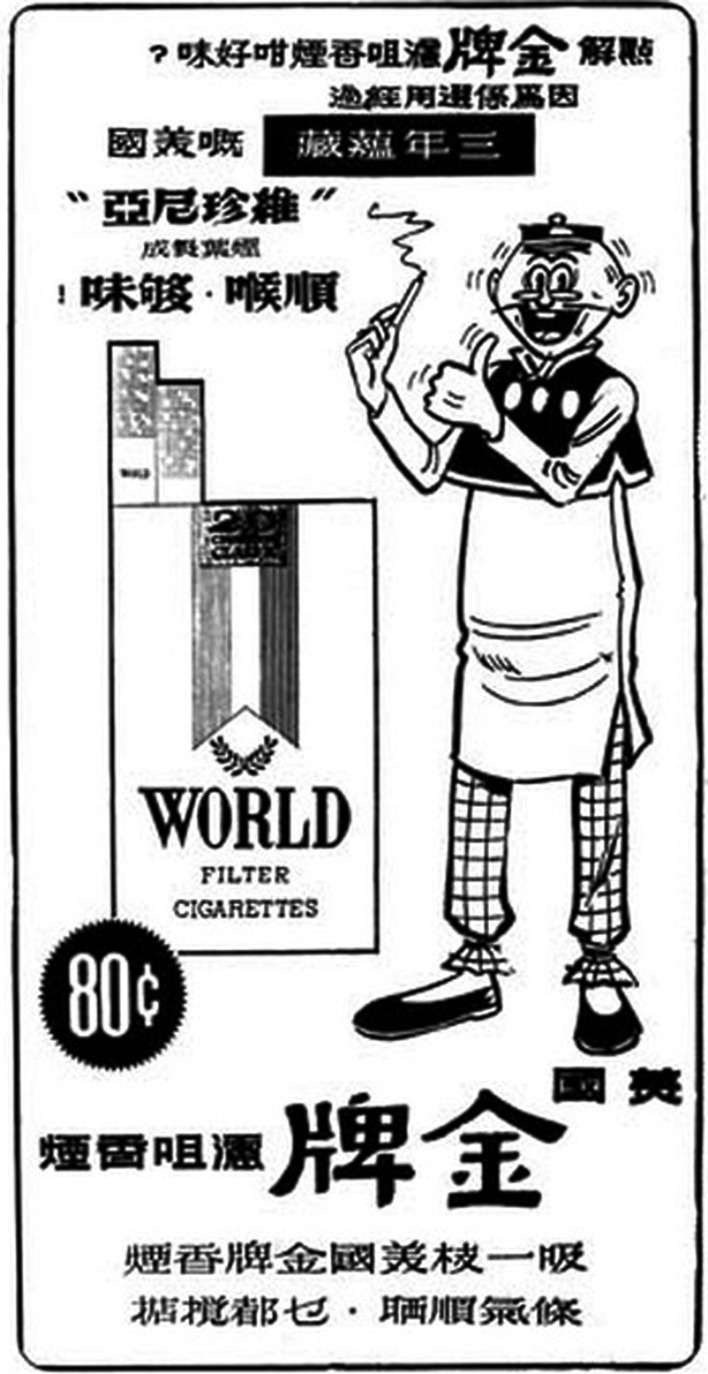
**Hong Kong newspaper advertisement from the 1950s for World Filter Cigarettes**. The Chinese script (written Cantonese) at the bottom of this advertisement for 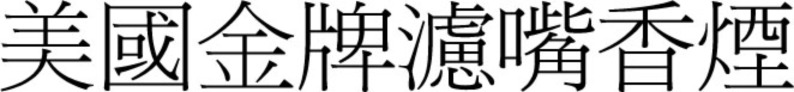 (*lit*. America Gold Brand filter cigarettes) is in the older RL direction. (Source: i.uwants.com/u/attachments/day_101118/20101118_ad66e669de9c3f1e16d90gyl0bEOwmZY.jpg; accessed 6th July 2012.)

**Figure 2:**
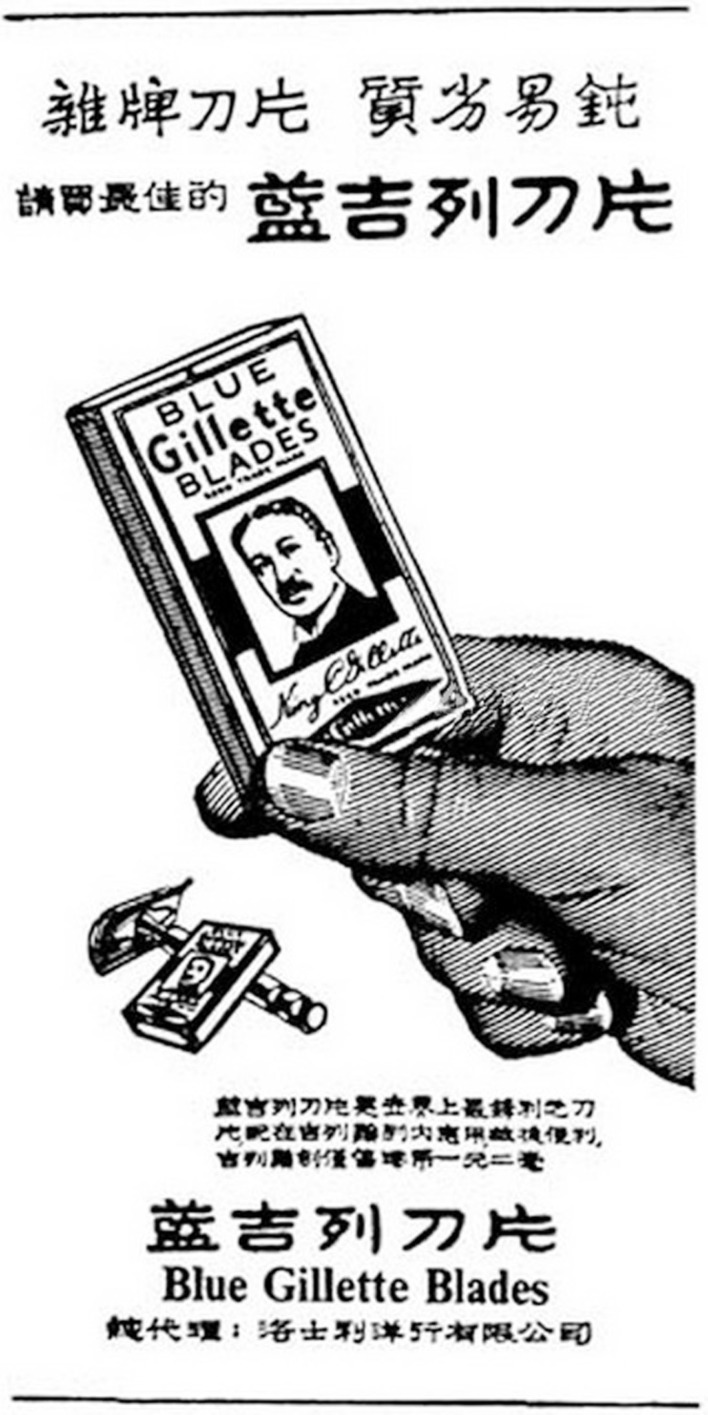
**Hong Kong newspaper advertisement from the 1950s for Blue Gillette Blades**. The Chinese script (Written Chinese) in this advertisement for 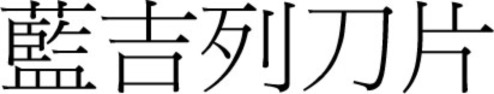 “Blue Gillette Blades” is in the newer LR direction. (Source: i.uwants.com/u/attachments/day_101216/20101216_9ba7ff0e5540629a4605Xkq70OFYAFHE.jpg; accessed 6th July 2012. Image is minimally altered.)

The Cantonese language and culture in Hong Kong and Macau provide an interesting test case. Cantonese is the majority language in both Hong Kong and Macau. The language, culture, and history of the Cantonese societies in Hong Kong and Macau are minimally different. Most written correspondence is conducted in Written Chinese (i.e., written Mandarin), but for informal communication, sometimes written Cantonese is used^2^. Both are written using Chinese script. Chinese script has witnessed the introduction of two new script directions in the last 100 years. Chinese is traditionally written in the vertical orientation from top to bottom (TB), and then the columns from right-to-left (RL). Under the influence of European scripts, horizontal orientation started to gain popularity around the 1920s, but it has never totally replaced the traditional TB direction. The dominant horizontal direction before the 1950s was from RL, but the left-to-right (LR) direction also existed. In the 1950s, the Mainland Chinese government adopted the LR script direction, while in Hong Kong and Macau both the LR and RL directions continued to coexist in the 1950s. Figures 1 and 2 below show two Hong Kong newspaper advertisements from the 1950s. In Figure 1, the Chinese script in the advertisement for World Filter Cigarettes, or 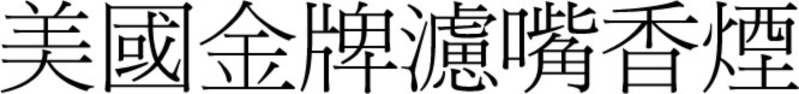 (*lit*. America Gold Brand filter cigarettes), is in the older RL direction. (For instance, notice the position of the question and exclamation marks at the left edge of the lines, corresponding with the end of a sentence in the RL direction.) In Figure 2, the Chinese script in the advertisement for 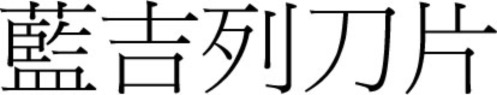 “Blue Gillette Blades” is in the newer LR direction (notice the location of the comma on the right, at the end of the sentence in the LR direction). Also notice that in Figure 1, the language used is written Cantonese, which gives a colloquial feeling, whereas Figure 2 uses Written Chinese, which is more formal in register. The newer LR direction might have been used to give the advertisement in Figure 2 a more formal, luxurious, and fashionable feeling^3^. (Note that all Chinese scripts in the running text of this paper are in the LR direction.)

**Figure 3:**
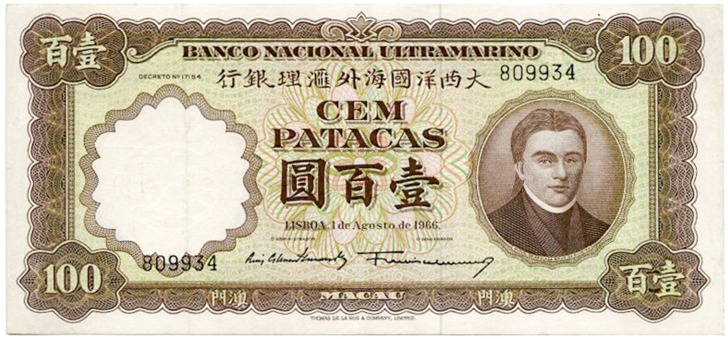
**The 1952 version of the 100 Macanese pataca banknote**. Both the Chinese name of the issuing bank 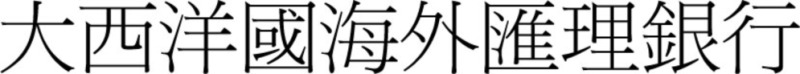 and the denomination 
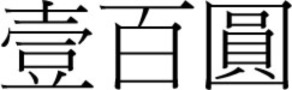
 were in the older RL direction. (Source: 2.bp.blogspot.com/_7RFtQJBFMYI/SjgQLMrj34I/AAAAAAAADE4/Eoh9UnWZzAc/s1600/macau100patacas1966.jpg; accessed 6th July 2012.)

**Figure 4:**
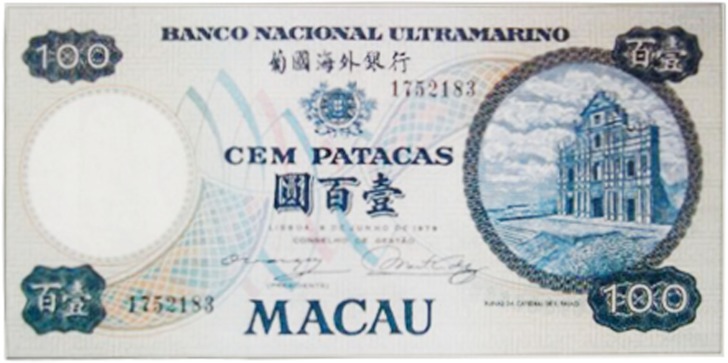
**The 1973 version of the 100 Macanese pataca banknote**. The Chinese name of the issuing bank 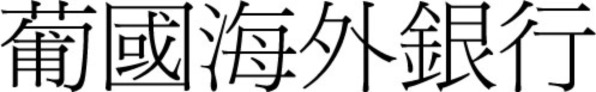 had changed to the newer LR direction, but the denomination 
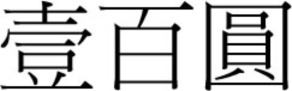
 “100 patacas” was in the older RL direction. (Source: www.banknote.ws/COLLECTION/countries/ASI/MAC/MAC0057ao.JPG; accessed 13th July 2012.)

**Figure 5:**
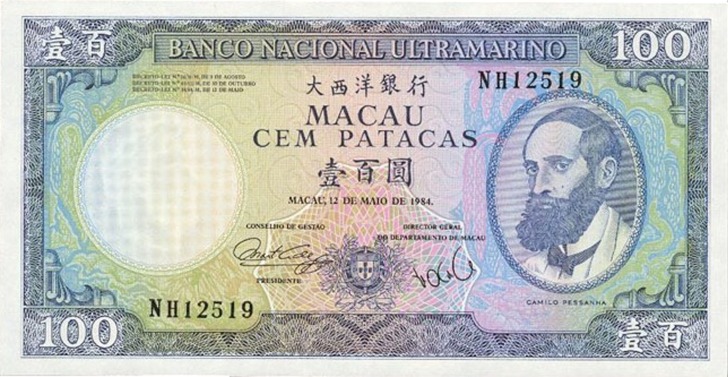
**The 1981 version of the 100 Macanese pataca banknote**. Both the Chinese name of the issuing bank 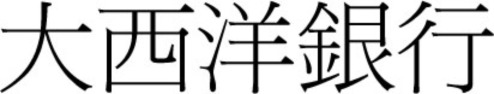 and the denomination 
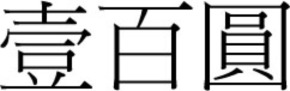
 “100 patacas” were in the newer LR direction. (Source: www.vincenzo.altervista.org/catalog/macao/mao061_f.jpg; accessed 6th July 2012.)

There was a gradual shift to the LR direction from sometime before the 1950s, and by the 1970s, the LR direction had become the dominant horizontal direction in Hong Kong and Macau. More evidence of this change in script direction is seen in the 1952, 1973, and 1981 versions of the 100 Macanese pataca banknote (“pataca” is Portuguese for “peso”), as shown in Figures 3–5 below. The Portuguese script with its LR script direction is the same on the three banknotes. At the top of the banknote is the name of the issuing bank *Banco Nacional Ultramarino*, and in the middle is the denomination *cem patacas* “100 patacas.” In 1952 version (Figure 3), both the Chinese name of the bank 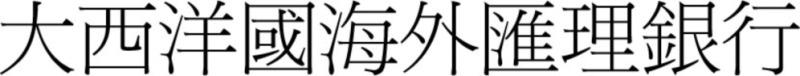 (*lit*. Overseas Banking Company of the Atlantic Country) and the denomination 
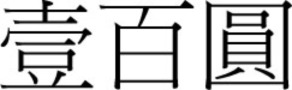
 “100 patacas” were in the older RL direction. Interestingly, in the 1973 version (Figure 4), the direction of the (new) Chinese name of the bank 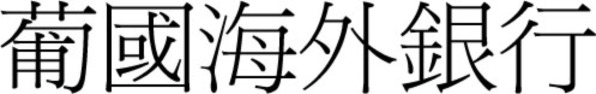 (*lit*. Overseas Bank of Portugal) had changed to the newer LR direction, but the denomination 
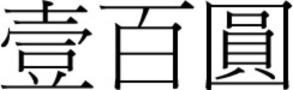
 “100 patacas” remained in the traditional RL direction. Finally, in the 1981 version (Figure 5), the changing of the script direction was complete; both the (current) name of the bank 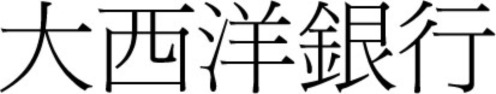 (*lit*. Bank of Atlantic Ocean) and the denomination 
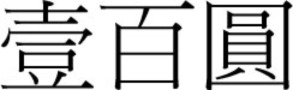
 “100 patacas” were in the LR direction.

**Figure 6:**
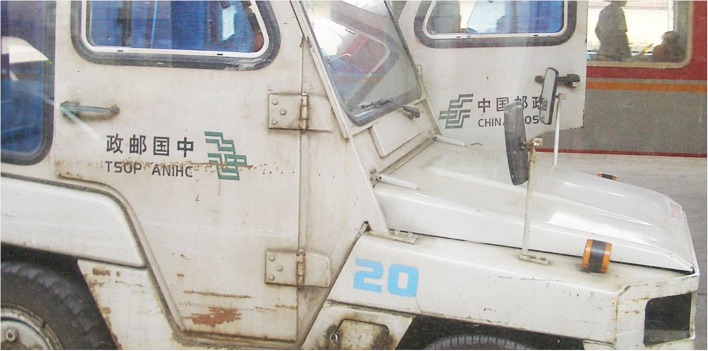
**Texts on a China Post vehicle**. The Chinese text 
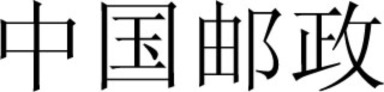
 and the English text CHINA POST on the starboard side of the vehicle (foreground) run in the RL direction, whereas the same texts on the port side run in the LR direction. (Source: upload.wikimedia.org/wikipedia/commons/7/7e/VM_5485_China_Post_Office_car_at_Zhengzhou_Train_Station.jpg; accessed 6th July 2012.)

Currently all three directions (TB, RL, LR) are found in Hong Kong and Macau. While LR is dominant, TB is still very often seen in publications. RL, however, is exceedingly rare. To sample the prevalence of the various script directions, news articles and photograph captions in Section A (12 pages) of Macao Daily News (a Chinese newspaper) were surveyed on 6th December 2010. Of the 64 news articles, 38 articles (59.4%) had LR headlines and 26 articles (40.6%) had TB headlines; 49 articles (76.6%) had LR body texts, and 15 articles (23.4%) had TB body texts^4^. Of the 50 photograph captions, 34 captions (68%) were LR, and 16 captions (32%) were TB. All these figures reflect the general trend that the LR direction is dominant, but a significant minority of texts still run in the traditional TB direction. The RL direction was not found in the newspaper. RL texts are rare in general and are primarily found in, for instance, public signs which are old or have an old theme. Sometimes they are also found on the starboard side of vehicles where the beginning of the line (the right hand side) corresponds with the front of the vehicle, as shown in Figure 6 below of a vehicle belonging to China Post in Mainland China.

In terms of school education in Hong Kong and Macau, in the first half of the twentieth century students were mostly required to write Chinese in the TB direction, and it was rare to handwrite Chinese in the horizontal orientation. However, people were accustomed to seeing horizontal writing in printed media. Nowadays, students at most schools in Hong Kong and Macau are required to write primarily in the LR direction, although the TB direction is still used sometimes, especially during Chinese lessons (for both pencil/ballpoint pen writing and ink brush calligraphy). Handwriting in the RL direction has always been rare. In the first half of the twentieth century when the RL direction was popular, it was primarily used in the printed media, and handwritten Chinese was most usually written in the TB direction. Literate people of all ages, including the participants in this study, have equal ease in handwriting in the TB and LR direction.

In the experiments described in the following section, if people’s conception of time is influenced by script direction, it would not be surprising if the participants who were literate before the 1950s demonstrate time in the RL direction at least some of the time. On the other hand, one would expect participants who became literate after the 1970s to demonstrate time mostly in the LR direction.

## Materials and Methods

The experiments conducted followed the guidelines and instructions described fully in Boroditsky et al. (2007, 2008)^5^. The following is a summary of the participants, settings, equipment, and procedures specific to the experiments conducted by the author.

### Participants

Ten participants were interviewed in July 2008: five in Hong Kong and five in Macau. All the participants were literate in Chinese, and had at least graduated from high school (except the youngest participant who was 15 years old at the time and was still at high school). All were native speakers of Standard Cantonese, and all had at least some competence in English and Mandarin. Three had parent(s) who were fluent in Teochew (another Sinitic language), and the Macau participants had learnt at least some Portuguese at school. The most relevant sociolinguistic factor to the data was age, and no other sociolinguistic factors (e.g., place of origin, gender) correlated with observable differences in the data. The participants can be divided into two cohorts based on age: the “older participants” were born in or before the 1950s (the average age was 67.3 years), and the “younger participants” were born in or after the 1970s (the average age was 26.7 years). Unfortunately, the age decomposition of the participants was skewed: of the 10 participants, only three were in the older cohort, while seven were in the younger cohort. (The difference in the results based on age was an unforeseen finding, and hence age was not properly controlled for.) Despite the difference in size of the two cohorts, there were clearly observable differences in the results of the two cohorts in this study.

### Settings

The tasks were conducted indoors at the participants’ homes, which were all in high-rise apartment blocks. (Single-family detached houses are rare in Hong Kong and Macau.) The participants were tested individually, and all the instructions were given verbally in Cantonese by the author. In anticipation of mostly horizontal results, efforts were made to minimize horizontal (and vertical) influences in the environment by positioning the participants on the edge of a round table or on the floor, diagonal to walls and tile patterns on the floor. The author positioned himself next to or behind the participants (randomly to the left or right), facing the same direction as them, so that the participants would not feel the need to adjust the direction of their presentation to the perspective of the author. Each of the tasks consisted of two sittings, conducted at least 30 min apart. The participants were not told about the second sitting until it was about to begin. In the second sitting, the participants were turned 180° (or 90° for two participants, due to the limitation in space) in relation to the direction they were facing in the first sitting.^6^

### Card arranging task

The kit for the card arranging task involved eight sets of round laminated photograph cards. Each set showed a different temporal progression, and each set had four cards, showing different stages of the temporary progression. The sets were divided into two groups: Group A and Group B. The following are the descriptions of the sets:

**Table:** 

Group A:	
banana:	a banana gradually being peeled and eaten
chicken:	a chick hatching from a brown egg
Cosby:	Bill Cosby at different ages
puppy:	a growing black puppy at different ages
Group B:	
green apple:	a green apple gradually being eaten
duck:	a duckling hatching from a white egg
grandpa:	Boroditsky’s grandfather at different ages
pregnant belly:	a woman’s belly growing through pregnancy

With the cards facing down and separated into the two groups, the participants were asked to randomly select one group; the selected group was used for the first sitting, and the other group was used for the second sitting. From the group of cards used for the session, the author selected a set of cards (of the same temporal progression), and presented them to the participants in random order. After the participants had looked at them, they were asked to arrange the cards in front of themselves in the correct chronology, from the earliest to the latest state. The participants were told that the aim was to see the chronology of the cards, when in fact the author’s primary interest was the spatial placement of the cards. The direction and orientation of the cards were then recorded on a coding sheet.

### Time-points task

The time-points task was conducted after the card arranging task. There were also two sittings. The author stood next to the participant, both facing the same direction. The author held a small token (e.g., nut, candy, marshmallow) in the air, immediately in front of the participant. The participant was then told that the token represented a moment in time, e.g., 
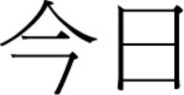
 *gam1 jat6* (now-day) “today.” This moment in time is called the “reference time point” (the reference time point is not necessarily current in relation to the time of testing). Next, the participant was given two further tokens of the same sort, and told that one represented a point in the past in relation to the reference time point, e.g., 
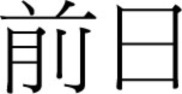
 *cin4 jat6* (front day) “day before yesterday,” and the other represented a time point in the future in relation to the reference time point, e.g., 
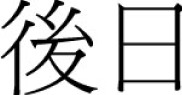
 *hau6 jat6* (back day) “day after tomorrow.” After that, with the reference time point token still held in the air by the author, the participant was asked to place the relative past and relative future tokens in the vicinity of the reference time point token, so that the three tokens represented the relative order of the three temporal expressions. The participants were asked seven sets of pre-selected temporal expressions in each setting. The fourteen sets of spatial expressions used in the time-points task is shown in Table 1. (The last set in each sitting was not mentioned in Boroditsky et al., 2007, 2008; they were added because they use the front-back metaphors, rather than the up-down (UD) metaphors more commonly used in Cantonese. The difference in metaphors turned out to not have any influence on the placements of the tokens.) The orientation and direction of the tokens were then recorded on a coding sheet.

**Table 1:** **The fourteen sets of temporal expressions used in the time-points task**.

Relative past time point	Reference time point	Relative future time point
**SITTING ONE**		
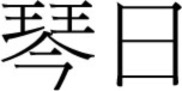 *kam4 jat6* “yesterday”	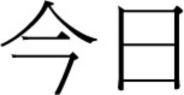 *gam1 jat6* “today”	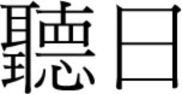 *ting1 jat6* “tomorrow”
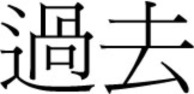 *gwo3 heoi3* “past”	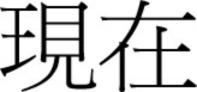 *jin6 zoi6* “present”	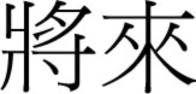 *zoeng1 loi4* “future”
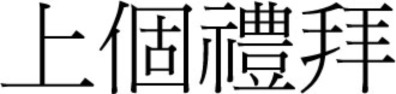 *soeng6 go3 lai5 baai3* “last week”	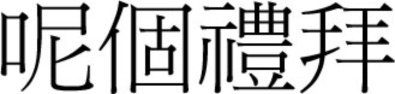 *ni1 go3 lai5 baai3* “this week”	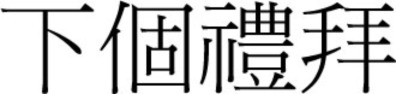 *haa6 go3 lai5 baai3* “next week”
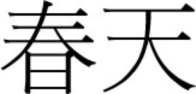 *ceon1 tin1* “spring”	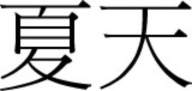 *haa6 tin1* “summer”	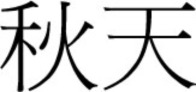 *cau1 tin1* “autumn”
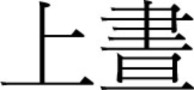 *soeng6 zau3* “morning/a.m.”	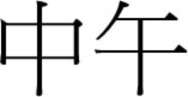 *zung1 ng5* “noon”	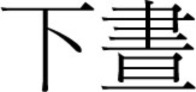 *haa6 zau3* “afternoon”
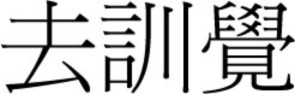 *heoi3 fan3 gaau3* “go to sleep”	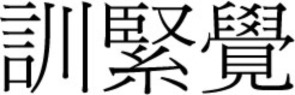 *fan3 gan2 gaau3* “sleeping”	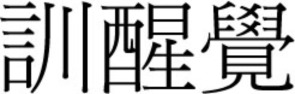 *fan3 seng2 gaau3* “wake up”
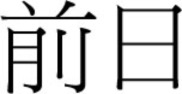 *cin4 jat6* “day before yesterday”	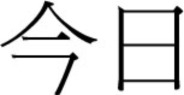 *gam1 jat6* “today”	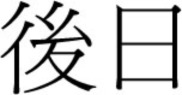 *hau6 jat6* “day after tomorrow”
**SITTING TWO**		
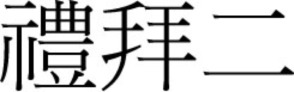 *lai5 baai3 ji6* “Tuesday”	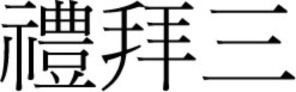 *lai5 baai3 saam1* “Wednesday”	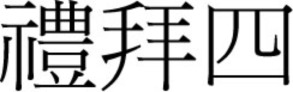 *lai5 baai3 sei3* “Thursday”
BB 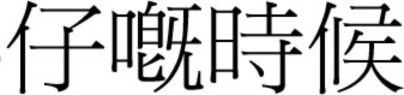 *bi4 bi1 zai2 ge3 si4hau6* “when one is a baby”	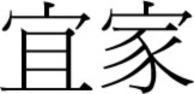 *ji4 gaa1* “now”	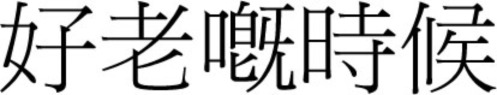 *hou2 lou5 ge3 si4 hau6* “when one is very old”
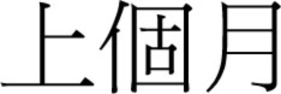 *soeng6 go3 jyut6* “last month”	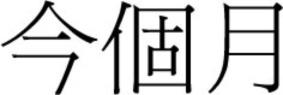 *gam1 go3 jyut6* “this month”	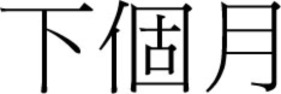 *haa6 go3 jyut6* “next month”
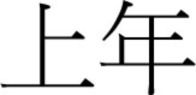 *soeng6 nin2* “last year”	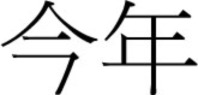 *gam1 nin4* “this year”	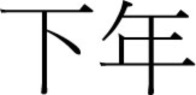 *haa6 nin2* “next year”
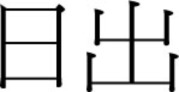 *jat6 ceot1* “sunrise”	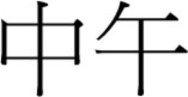 *zung1 ng5* “noon”	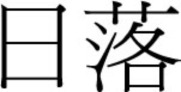 *jat6 lok6* “sunset”
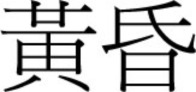 *wong4 fan1* “dusk”	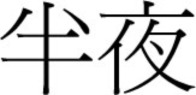 *bun3 je6* “midnight”	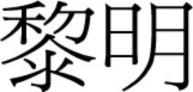 *lai4 ming4* “dawn”
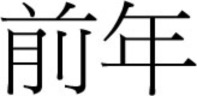 *cin4 nin2* “year before last”	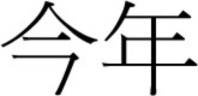 *gam1 nin4* “this year”	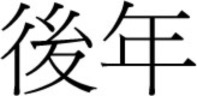 *hau6 nin2* “year after next”

## Results

For the card arranging task, there were three types of arrangements: RL (i.e., the earliest card in the extreme right), LR, and the “LR diamond” pattern which was used consistently by participant C (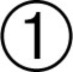 is the earliest card, e.g., “an entire banana,” whereas 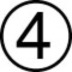 is the latest card, e.g., “only the banana peel is left”):


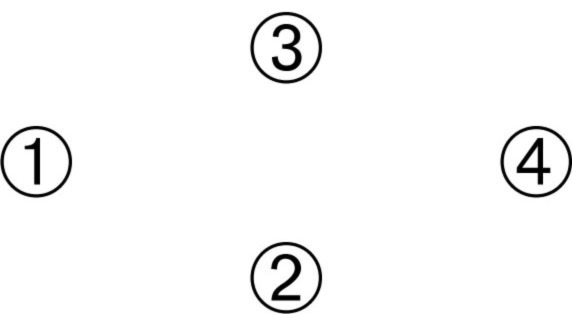


The results of the card arranging task are shown in Table 2.

**Table 2:** **Frequency of arrangement direction in the card arranging task**.

Participant ID	Age	RL	LR	LR diamond
A	73	8		
B	69	8		
C	60			8
D	35	8		
E	28		8	
F	28	7	1	
G	28		8	
H	27		8	
I	26		8	
J	15		8	

*For participant F, the set of cards that were placed in a LR direction was the “duck” set. No reason was given for the discrepancy in the direction of the cards*.

For the time-points task, there were five types of arrangements: RL, LR, UD, down-up (DU), and back-front (BF; back being behind one’s shoulders). One older participant (participant A) and one younger participant (participant D) did not participate in the time-points task. The following table summarizes the results of the time-points task.

For participant B, the pair of words that she indicated in the DU direction was 
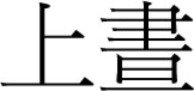
 *soeng6 zau3* (up noon) “morning/a.m.” and 
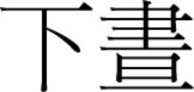
 *haa6 zau3* (down noon) “afternoon,” which interestingly contradicted the temporal metaphors in those terms. The pair of words that she indicated in the BF direction was 
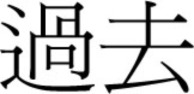
 *gwo3 heoi3* (pass go) “past” and 
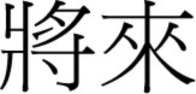
 *zoeng1 loi4* (will come) “future”; this is perhaps a Europeanized way of gesturing past and future. All other sets of words were gestured in the same RL direction. The results of the time-points task are shown in Table 3.

**Table 3:** **Frequency of arrangement direction in the time-points task**.

Participant ID	Age	RL	LR	UD	DU	BF
B	69	12			1	1
C	60		14			
E	28		14			
F	28			14		
G	28		14			
H	27		14			
I	26		14			
J	15		14			

The proportion of RL results for the two tasks combined is shown in Table 4.

**Table 4:** **Proportion of RL responses**.

Participant ID	Age	Proportion of RL responses
A	73	1
B	69	0.9091
C	60	0
D	35	1
E	28	0
F	28	0.3182
G	28	0
H	27	0
I	26	0
J	15	0

*The correlation between age and the proportion of RL responses is statistically significant (one-tailed, 0.05 level of significance): *r*(*n* = 9) = 0.64, *p* = 0.03*.

## Analyses and Discussion

Despite the fact that the sample size was small, and the sizes of the cohorts were biased, the results from these experiments do suggest that script direction influences people’s spatial conception of time. The two oldest participants represented time consistently in a RL manner (except for two instances), and five of the seven younger participants produced LR results exclusively. This is consistent with the dominant horizontal script direction of Chinese that each cohort first learned: RL before the 1950s, and LR in and after the 1970s. Countering the general trend, three participants demonstrated time in the opposite horizontal direction or the UD direction. This is not too surprising, as all participants would have had experience in reading Chinese scripts in all of these various script directions (with UD considered to be the three-dimensional equivalent of TB). Very few results were in directions that did not match any conventional script directions: the “LR diamond” direction used by participant C in the card arranging task, and the DU and BF directions (one instance each) used by participant B in the time-points task^7^.

A number of deficiencies concerning the equipment and procedures of the experiments was discovered. The card arranging task itself might be biased in creating results which resemble script direction for literate speakers (at least in cultures where absolute frame of reference is not dominant), as the pictorial cards are visual representation of events, similar to how writing is a visual means of representing language. Nonetheless, a possible counterexample to this claim is the lack of TB results in this study: if the pictorial cards are analogous to writing, one would expect there to be some TB results with the card arranging task. The reason may be due to human anatomy. The cards were about the same size as the participants’ palms, and if the four cards were to be placed linearly, then placing them in a LR or RL direction on a table or on the floor, or placing them in a UD or DU direction on a standing whiteboard with the help of magnets, is relatively effortless. However, placing the four cards in a TB or BT direction on a table, or on the floor, requires extension of the arm if the participant is seated in front of a table, or the upper body has to lean forward if the participant is seated on the floor. A suggestion for future experiments is to have the participants put the cards on a standing whiteboard or other vertical surface placed in front of them.

It was also perhaps less optimal to conduct the time-points task immediately after the card arranging task, as the card arranging task was perhaps biased toward horizontal results, and might have primed the participant to provide results in the same directions when performing the time-points task.

There are several ways the hypothesis can be tested further. In addition to having a greater number of literate participants across various age groups, having control groups of illiterate speakers across various age groups would be essential (albeit younger illiterate speakers might be harder to find in Hong Kong and Macau). Coordinated large-scale international investigations should be conducted. In the region, Taiwan and Japan also have a similar mix of text in TB and LR directions. Impressionistically, the rate of use of TB is higher in Taiwan and Japan than in Hong Kong and Macau, so perhaps the rate of TB results would also be higher in Taiwan and Japan. Conversely, the rate of TB usage is low in Korea and Mainland China nowadays, so the rate of TB results would presumably be lower in Korea and Mainland China. In the region, there are also the interesting cases of Mongolian, Uyghur, Panjabi, and Hindi-Urdu. Mongolian in China is written in the traditional Mongol script, which runs only in the TB direction, whereas Mongolian in Mongolia is written in Cyrillic script, which runs in the LR direction. Uyghur in China is written in Perso-Arabic script, which runs in the RL direction, whereas Uyghur communities in places like Kazakhstan, Kyrgyzstan, and Turkey commonly write Uyghur in LR scripts like Cyrillic and/or Roman scripts. Panjabi in Pakistan is written in the RL Perso-Arabic script (“Shahmukhi”), whereas Panjabi in India is written in the LR Gurmukhi script (and sometimes in the LR Devanagari script). Hindi and Urdu are very similar languages, with Urdu written in the RL Perso-Arabic script, and Hindi in the LR Devanagari script. Coordinated experiments on these various speech communities would no doubt add valuable data to this debate.

## Conclusion

To conclude, the results of the two experiments suggest that script direction affects literate speakers’ conception of time. The older participants in this study predominantly demonstrated time progression in the RL direction, while the younger participants predominantly demonstrated time progression in the LR direction, consistent with the dominant horizontal direction of Chinese script at the time they started to become literate (RL before the 1950s and LR after the 1970s in Hong Kong and Macau). It is interesting that after at least 40 years of dominance of the LR script direction, some of the oldest participants still demonstrated time using the older RL direction.

## Conflict of Interest Statement

The author declares that the research was conducted in the absence of any commercial or financial relationships that could be construed as a potential conflict of interest.

## Footnotes

^1^This investigation is conducted under the “Time in Space” project (Boroditsky et al., 2007, 2008), the aims of which are to investigate the variation in how time is conceptualized across languages and cultures, and whether the way space is conceptualized affects the way time is conceptualized, in particular how the variation in the dominant spatial frames of reference (Levinson, 2003) in a culture affect how people conceptualize time.

^2^(Modern) Written Chinese is a standardized written language based on formal spoken Mandarin, and hence “written” is capitalized for Written Chinese. On the other hand, there is no standardized written variety of Cantonese, and hence “written” in written Cantonese is not capitalized. When Cantonese is written, it is usually a direct written version of spoken Cantonese. For words with no cognates in Mandarin, they are represented using various *ad hoc* methods, sometimes including Roman alphabets. For instance, *leng3-di1* (beautiful-comparative) ‘more beautiful’ may be written as  or D. See Snow (2004) on written Cantonese.

^3^The correlation of the advertisement in written Cantonese having the RL direction, and the advertisement in Written Chinese (i.e. Written Mandarin) having the LR direction is indirect. During the 1950s, advertisements for cheaper and/or traditional items correlated with the traditional RL direction, and this type of advertisements for cheaper items may use written Cantonese rather than Written Chinese. Advertisements for more expensive and/or Western items correlated with the newer LR direction, and advertisements for expensive items are always written in Written Chinese.

^4^The script direction of the titles need not correspond to the script direction of the body texts. Out of the 49 (76.6%) articles with LR body texts, 33 articles have LR headlines (51.6%), and 16 articles (25%) have TB headlines (to the left of the body text); out of the 15 articles (23.4%) with TB body texts, 10 articles (15.6%) have TB headlines, and 5 articles (7.8%) have LR headlines (on top of the body text).

^5^Electronic versions of these can be found in http://fieldmanuals.mpi.nl/

^6^The difference in the facing direction for the two sittings was aimed at determining whether people think in absolute frame of reference. (For instance, Boroditsky and Gaby (2010) report that their Kuuk Thayoore participants frequently demonstrated time in the east to west direction, no matter which direction they were facing.) None of the Cantonese participants utilized absolute frame of reference in the tasks; the majority of responses were LR or RL. Cantonese primarily utilizes intrinsic frame of reference for objects that are thought to have one (non-vertical facing) face, and relative frame of reference for other objects.

^7^On the other hand, spatial metaphors of time appeared to have little effect on the way the participants represented time in the time-points task. Except for participant B, all participants consistently demonstrated time in one single direction, no matter which axes the spatial metaphors were based on. For instance, most participants demonstrated  *soeng6 nin2* (up year) ‘last year’ versus  *haa6 nin2* (down year) ‘next year’, and  *cin4 nin2* (front year) ‘year before last’ versus  *hau6 nin2* (back year) ‘year after next’, in the LR or RL direction uniformly. Moreover, the horizontal axis is not used in spatial metaphors of time in Cantonese. Instances where the participants utilized the vertical or sagittal axes–axes on which the spatial metaphors of time in Cantonese exist – were rare. Interestingly, there is one instance (participant B) where  *soeng6 zau3* (up noon) ‘morning/a.m.’ versus  *haa6 zau3* (down noon) ‘afternoon’ were demonstrated in the DU direction, opposite to that indicated by the spatial metaphors. In gestural studies, it is also claimed that unconscious gestures of time are often in axes different from the ones used by spatial metaphors of time (e.g., Casasanto and Jasmin, 2012, for gestures by English speakers).

## References

[B1] Bergen B. Chan Lau T. (2012). Writing direction affects how people map space onto time. Front. Psychol. 3:109 10.3389/fpsyg.2012.00109 22514546 PMC3322406

[B2] Boroditsky L. (2000). Metaphoric structuring: understanding time through spatial metaphors. Cognition 75, 1–28 10.1016/S0010-0277(99)00078-5 10815775

[B3] Boroditsky L. Gaby A. (2010). Remembrances of times east: absolute spatial representations of time in an Australian Aboriginal community. Psychol. Sci. 21, 1635–1639 10.1177/0956797610386621 20959511

[B4] Boroditsky L. Gaby A. Levinson S. (2007). “Time in space,” in Language & Cognition Group Field Manual, Vol. 10, ed. Majid A. (Nijmegen: Max Planck Institute for Psycholinguistics), 59–80

[B5] Boroditsky L. Gaby A. Levinson S. (2008). “Time in space,” in Language & Cognition Group Field Manual, Vol. 11, ed. Majid A. (Nijmegen: Max Planck Institute for Psycholinguistics), 52–76

[B6] Boroditsky L. Ramscar M. (2002). The roles of body and mind in abstract thought. Psychol. Sci. 13, 185–188 10.1111/1467-9280.00434 11934006

[B7] Casasanto D. Boroditsky L. (2008). Time in the mind: using space to think about time. Cognition 106, 579–593 10.1016/j.cognition.2007.03.004 17509553

[B8] Casasanto D. Bottini R. (2010). “Can mirror-reading reverse the flow of time?” in Spatial Cognition VII, eds Hölscher C. Shipley T. F. Olivetti Belardinelli M. Bateman J. A. Newcombe N. S. (Berlin: Springer), 335–345

[B9] Casasanto D. Jasmin K. (2012). The hands of time: temporal gestures in English speakers. Cognit. Ling. 23 (in press).

[B10] Casasanto D. Lozano S. (2006). “Metaphor in the mind and hands,” in Proceedings of 28th Annual Conference of the Cognitive Science Society, eds Sun R. Miyake N. (Mahwah: Lawrence Erlbaum Associates), 142–147

[B11] de Sousa H. (2006). What is switch-reference? From the viewpoint of the young people’s switch-reference system in Menggwa Dla. Te Reo 49, 39–71

[B12] Fuhrman O. Boroditsky L. (2010). Cross-cultural differences in mental representations of time: evidence from an implicit nonlinguistic task. Cogn. Sci. 34, 1430–1451 10.1111/j.1551-6709.2010.01105.x 21564254

[B13] Gibbs R. W. Jr. (1994). The Poetics of Mind: Figurative Thought, Language, and Understanding. New York: Cambridge University Press

[B14] Lakoff G. Johnson M. (1980). Metaphors We Live By. Chicago: University of Chicago Press

[B15] Levinson S. (2003). Space in Language and Cognition. Cambridge: Cambridge University Press

[B16] Moore K. (2006). Space-to-time mappings and temporal concepts. Cognit. Ling. 17, 199–244 10.1515/COG.2006.005

[B17] Núñez R. E. Sweetser E. (2006). With the future behind them: convergent evidence from Aymara language and gesture in the crosslinguistic comparison of spatial construals of time. Cogn. Sci. 30, 401–450 10.1207/s15516709cog0000_62 21702821

[B18] Snow D. (2004). Cantonese as a Written Language. Hong Kong: Hong Kong University Press

[B19] Traugott E. C. (1978). “On the expression of spatio-temporal relations in language,” in Universals of Human Language, Vol. III, eds Greenberg J. H. Ferguson C. A. Moravcsik E. A. (Stanford: Stanford University Press), 369–400

[B20] Tversky B. Kugelmass S. Winter A. (1991). Cross-cultural and developmental trends in graphic productions. Cogn. Psychol. 23, 515–557 10.1016/0010-0285(91)90005-9

